# Valorization of a Low-Pulp Brazilian Native Fruit Pitomba (*Talisia esculenta* Radlk) Through the Production of Nutritive Powders with the Whole Fruit

**DOI:** 10.1007/s11130-026-01493-1

**Published:** 2026-04-21

**Authors:** Wanderson dos Santos Carneiro, Carlos Eduardo de Farias Silva, Kaciane Andreola, Ana Silvia Prata

**Affiliations:** 1 Technology Center, Federal University of Alagoas, Maceió, 57072- 970 Alagoas Brazil; 2 Institute of Food Technology, Campinas, São Paulo, 13070- 178 Brazil; 3 Department of Food Engineering and Technology, School of Food Engineering (FEA), State University of Campinas (UNICAMP), Campinas, São Paulo, 13083-862 Brazil

**Keywords:** Dietary fiber, Mineral profile, Brazilian biodiversity, Underutilized fruit

## Abstract

This study aimed to characterize powders obtained from pitomba (*Talisia esculenta* Radlk) which were prepared with pulp and seed (FA), seed (FB), and peel (FC) fractions in terms of physical, chemical and technological attributes to evaluate their potential for food applications. Proximal composition, minerals and functional–technological properties were determined. The powders presented low moisture content (4.88–10.67%) and water activity below 0.6. Protein levels ranged from 9.48% to 10.84%, while carbohydrates represented the major component. Ash content reached up to 5.26% in peel powder (FC), indicating a high mineral fraction and also exhibited the highest total dietary fiber content among the samples. Potassium was the predominant mineral (692.56–1578.23 mg/100 g), followed by calcium (62.01–354.35 mg/100 g) and magnesium (64.95–91.83 mg/100 g). When expressed as percentage of Dietary Reference Intakes (DRIs), peel powder (FC) provided up to 60.7% of the potassium requirement for adult women (19–50 years) and up to 68.6% for children (4–8 years), while also contributing substantially to calcium and magnesium intake. These results indicated that pitomba powders may serve as nutrient-dense ingredients for food formulations aimed at improving mineral intake. Functional–technological properties revealed that all powders displayed gelation ability and absorption capacities, with water absorption (2.49–3.01 g/g) consistently higher than oil absorption (1.83–2.21 g/g), and pH-dependent solubility, indicating adaptability to different formulation conditions. Together, the nutritional and technological attributes support their use in composite formulations while valorize a Brazilian native fruit.

**Supplementary Information:**

The online version contains supplementary material available at 10.1007/s11130-026-01493-1.

## Introduction

Brazil is recognized as one of the most biodiverse countries in the world, hosting three major biomes located in the tropical region. This natural diversity is reflected in the vast variety of native fruits, as well as in the significant number of introduced exotic species that have successfully adapted to the tropical climate [1].

A wide availability of underexplored species open an opportunity to transform natural resources into ingredients with potential industrial applications. Moreover, morphological components such as peels, seeds, and residual pulp are still largely discarded, representing a valuable opportunity for added value creation.

**Fig. 1:**
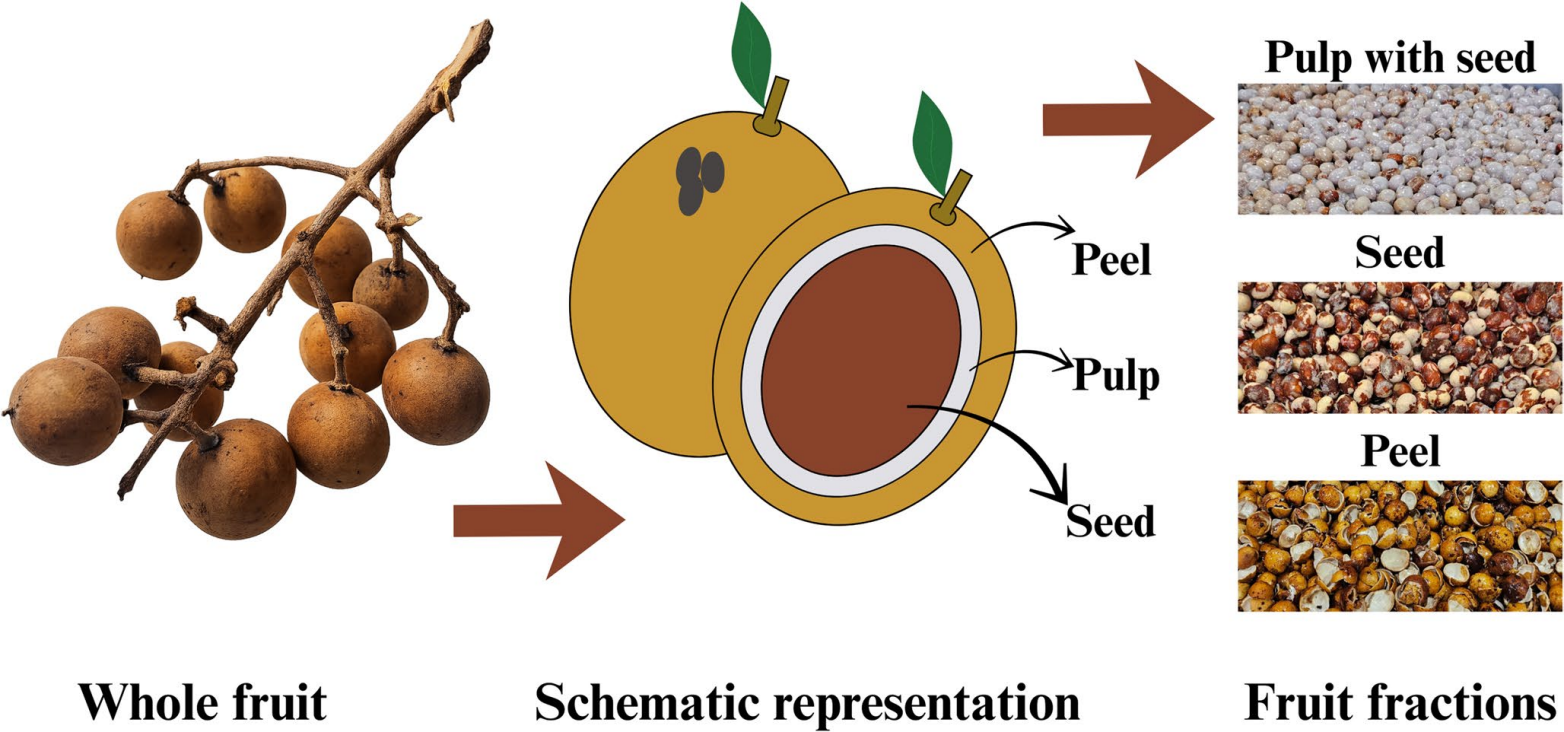
Morphological fractions of *Talisia esculenta* used in this study. From left to right: whole fruit, schematic anatomical representation, and separated fractions—pulp with seed, seed, and peel

Among these species, pitomba (*Talisia esculenta*) stands out as a native tropical fruit that ripens from January to March and is traditionally consumed on a seasonal basis in North and Northeast regions of Brazil. The fruit measures approximately 2 cm in diameter, with similar aspect of the lychee (*Litchi chinensis* Sonn.). The pulp represents about 15% of the fresh fruit by weight, while the peel (≈ 45%) and the seed (≈ 40%) together constitute the fruit majority, resulting in a considerable amount of waste [2]. The division of the fruit is illustrated in Fig. 1. Moreover, despite the pitomba being scarcely studied from a scientific perspective, particularly regarding the characterization of its different fractions, some punctual contributions indicate beneficial properties of its fractions. The seeds, for example, are popularly considered antidiarrheal and used as an astringent material [3].

In the broader context of agricultural and food processing chains, plant residues such as peels, seeds, and other non-primary fractions are often underutilized. These materials can be considered by-products, as they are not the main commercial output but a result of processing steps or consumption practices [4]. The conversion of these residues into powders represents a viable alternative to promote the physicochemical stability of the material, facilitate the storage and transportation, and enhance their technological potential in food formulations [5, 6]. The drying and milling of these by-products not only reduce water activity and microbiological risk, but also increase the surface area of the solids [7]. Moreover, depending on their properties, the powders can be used to develop bioplastics [5], to extract molecules of interest such as enzymes, vitamins, antioxidants [8], and incorporation into different food systems, such as doughs, baked goods, powdered beverages, and supplements, contributing to the development of functional foods [6].

Although few studies have investigated the potential of pitomba fractions, some relevant advances have been reported. Fraga et al [2] characterized the pulp and peel, highlighting their mineral and carotenoid contents, Castro et al [9] isolated starch from the seeds and demonstrated its application as a thickener in ketchup; and Souza et al [10] extracted phenolic compounds from peel and seeds using eutectic solvents (specifically choline chloride–lactic acid, choline chloride–levulinic acid, choline chloride–glycerol, and L-proline–levulinic acid), obtaining fractions with significant antioxidant capacity. Although some advances have been made in the study of pitomba fractions, the available information is fragmented and restricted to isolated components or specific extraction processes.

Given the potential for the comprehensive utilization of pitomba, it is essential to examine the compositional and technological properties of each morphological fraction—including peel, seed, and pulp-with-seed. A separate extraction and characterization of these components provide a more detailed understanding of their technological and compositional attributes, supporting the design of targeted formulation strategies for the development of sustainable food products. This approach also promotes an efficient use of plant resources and contributes to sustainability initiatives within the food supply chain, while recognizing the value of regional biodiversity [11].

This study aimed to provide a systematic characterization of powders obtained from the morphological fractions (pulp and seed, seed, and peel) of pitomba (*Talisia esculenta*), with emphasis on their macro- and micronutrient composition and mineral contribution to Dietary Reference Intakes (DRIs). The study further evaluated functional–technological properties relevant to food formulation in order to assess their potential as nutrient-dense alternative ingredients.

## Materials and methods

Supplementary material incorporated the section of Materials and methods.

## Results and Discussion

### Visual Aspect and Color Analysis

**Fig. 2:**
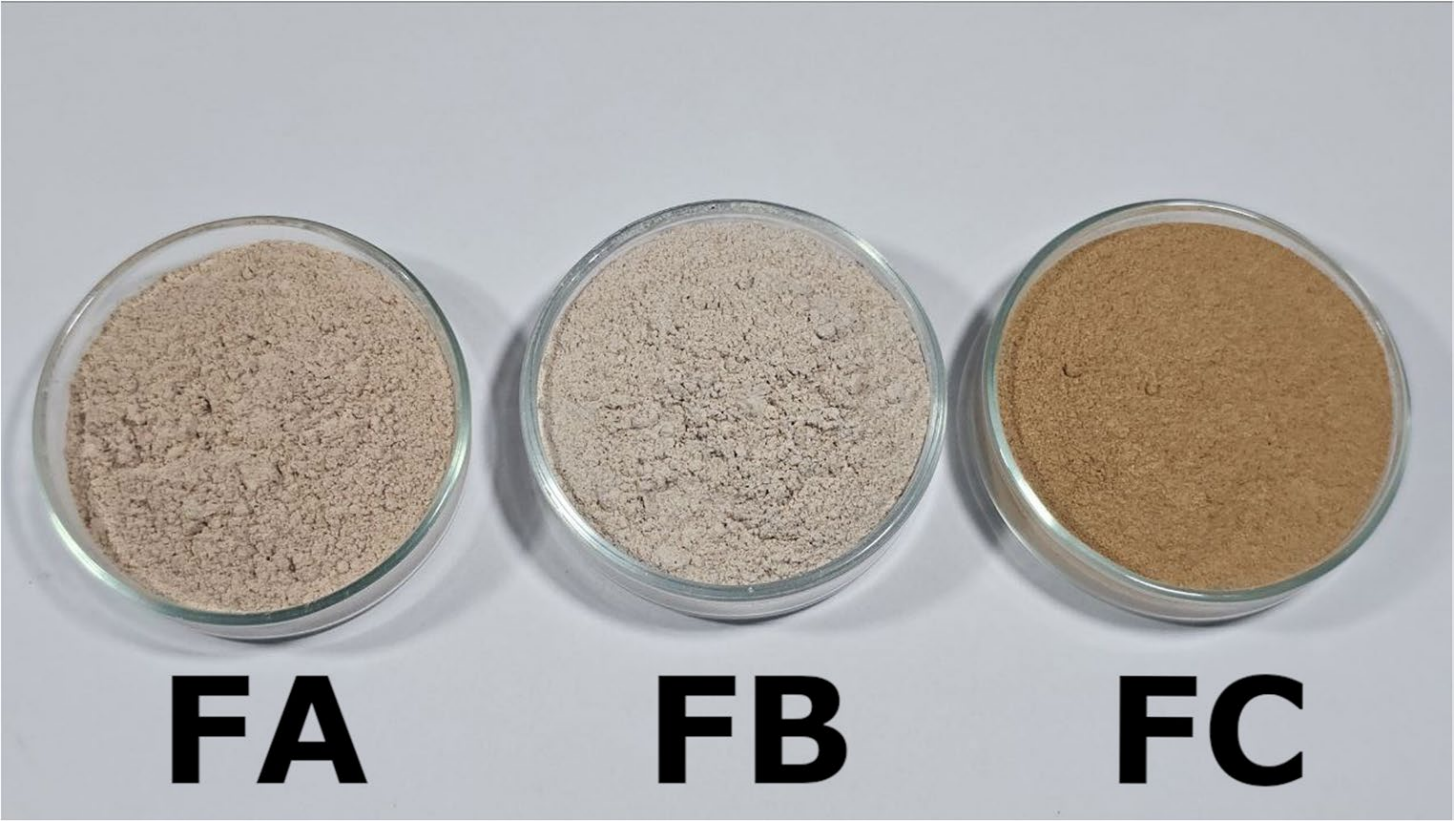
Powders produced from (FA) pulp and seed mixture, (FB) seed, and (FC) peel of Pitomba

The images of the powders obtained from pitomba fruit, highlighting visual differences among the samples are presented in Fig. 2. The coloration ranged from lighter to darker shades, which may be associated with the specific composition of each fraction and the thermal processing applied. These variations were confirmed by instrumental color analyses (Table 1), showing differences in luminosity (L*) values and in a* (redness tendency) and b* (yellowness tendency) parameters. In biochemical terms, the darker coloration of the peel powder (FC), evidenced by its lower L* value, can be attributed to the natural pigmentation of pitomba peel and to the tendency of fruit peels to undergo enzymatic and non-enzymatic browning during drying. Such reactions, including polyphenol oxidation and Maillard-type pathways, and can potentially contribute to the degradation or transformation of thermosensitive compounds such as flavonoids, vitamin C, and carotenoids, are widely reported in thermally processed fruit matrices [12]. In contrast, the seed powder (FB) exhibited the highest L* value and the lowest a* and b* values, consistent with the typically lower levels of sugars and pigments in seeds, which limit browning reactions. The pulp with seed powder (FA) showed intermediate color values, likely reflecting the presence of residual pulp components, including sugars capable of participating in mild Maillard reactions during drying [13]. Such differences may directly impact the visual acceptance of food products formulated with these powders, as observed in previous studies, where visual changes due to the incorporation of alternative powders positively or negatively affected sensory acceptance compared to control samples [14]. From an application perspective, this darker tone may be advantageous in products where a brown color is desirable, such as whole-grain bakery items, cereal bars, or meat-based formulations, where darker powders are often associated with higher fiber or mineral content.

**Table 1:** Color parameters (CIE L*a*b* system) of powders obtained from different parts of *Talisia esculenta* (pitomba)

Samples	L*	a*	b*	Comparison	ΔE*
FA	70.22 ± 0.76^b^	9.41 ± 0.04^b^	19.54 ± 0.26^b^	FA–FB	7.58 ± 0.09^c^
FB	74.98 ± 1.48^a^	6.36 ± 0.11^c^	15.91 ± 0.35^c^	FA–FC	20.70 ± 0.29^b^
FC	58.33 ± 1.19^c^	14.06 ± 0.12^a^	36.44 ± 0.17^a^	FB–FC	27.54 ± 0.10^a^

Each value is expressed as mean ± SD for a sample size (*n* = 3). Different letters indicate statistically significant differences among samples according to the applied *post hoc* test (Tukey’s HSD or Dunn’s test, p < 0.05)

ΔE* values were calculated according to the CIE76 equation using L*, a*, and b* measurements

The ΔE* value between FA and FB was 7.58, while markedly higher differences were observed for FA–FC (20.70) and FB–FC (27.54). All values exceeded the commonly reported perceptibility threshold (ΔE* > 3), confirming clear visual distinctions among the fractions. The results demonstrate that the peel fraction (FC) presents a substantially distinct color profile compared to FA and FB.

## Physical and Chemical Characterization

The values obtained from the physicochemical analyses of the powders are shown in Table 2. The final moisture content ranged from 4.88 to 10.67%. According to Resolution RDC No. 263, dated September 22, 2005, issued by ANVISA (Brazilian Health Regulatory Agency), the maximum allowable moisture content for commercialized flour and brans is 15.0% [15]. Although this reference pertains to wheat powder, it is commonly used as benchmark in the literature to evaluate the technological and regulatory acceptability of alternative plant-based powders. Therefore, the moisture levels of all pitomba powder samples were in full compliance with accepted safety and quality standards. As highlighted by Soarez and Souza [16], high moisture levels may lead to undesirable changes in texture and reduce the shelf life of powders, in addition to compromising their rheological properties and applicability in bakery products.

The powder obtained from the pulp and seed (FA) exhibited the highest protein content (10.84%). The values were similar to some data available in literature for peel of 9.31% (oven drying) and 10.51% (microwave drying), and seed of 9.76% (oven drying) and 8.01% (microwave drying) [17]. In contrast, the values in this study were higher than those reported by the study of Santos et al [18], which determined the proximate protein composition of pitomba peel during drying kinetics at three different temperatures: 50 °C (3.63 g/100 g), 60 °C (3.56 g/100 g), and 70 °C (3.44 g/100 g). Although both studies included drying at 50 °C, Santos et al. [18] also evaluated higher temperatures (60 and 70 °C), which may have influenced matrix behavior during dehydration. Nevertheless, differences in drying time are more likely associated with intrinsic biological variability of the raw material, including fruit maturity stage, seasonal and geographic factors, as well as structural differences in the morphological fractions and their processing mode. Small variations in tissue composition, pulp adherence to the peel fraction or seed presence can substantially affect the relation between macrocomponents in plant matrices.

**Table 2:** Physical and chemical composition of pitomba powders. Pulp with seed (FA), seed (FB), and peel (FC)

Parameters	Samples			
	FA	FB	FC	
Moisture content (%)	10.67 ± 0.05^a^	7.96 ± 0.04^b^	4.88 ± 0.03^c^	
Proteins (%)	10.84 ± 0.19^a^	10.26 ± 0.10^b^	9.48 ± 0.15^c^	
Ash (%)	2.62 ± 0.02^b^	2.36 ± 0.03^c^	5.26 ± 0.04^a^	
Lipids (%)	0.89 ± 0.08^a,b,c^	0.79 ± 0.09^a,b,c^	1.03 ± 0.09^a,b^	
a_W_ (-)	0.4856 ± 0.01^a^	0.3387 ± 0.01^b^	0.2601 ± 0.01^c^	
pH (-)	6.07 ± 0.07^b^	6.36 ± 0.05^a^	5.38 ± 0.07^c^	
Carbohydrates (%)	85.64 ± 0.19^b^	86.59 ± 0.18^a^	84.23 ± 0.03^c^	
Total dietary fiber (%)	22.25 ± 0.29^b^	20.88 ± 0.44^c^	71.44 ± 0.38^a^	
Caloric value (kcal/100 g)	401.41 ± 0.39^a,b^	402.23 ± 0.45^a,b^	391.48 ± 0.25^c^	
TTA (g acid latic/100 g)	1.46 ± 0.04^a,b,c^	1.27 ± 0.16^a,b,c^	1.51 ± 0.07^a,b,c^	
Total Soluble Solids - TSS (°Brix)	1.98 ± 0.03^a,b^	1.71 ± 0.01^c^	1.98 ± 0.03^a,b^	

Each value is expressed as mean ± SD for a sample size (*n* = 3). Different letters indicate statistically significant differences among samples according to the applied post hoc test (Tukey’s HSD or Dunn’s test, *p* < 0.05). Proximate components expressed on a dry basis (% = g/100 g), except for moisture content, which is expressed on a wet basis

The ash contents observed in the powders were consistent with values reported in the literature for tropical fruit by-products and even exceeded the maximum limit established (a reference value) by Normative Instruction No. 08/2005 for wheat powder (up to 2.5%) [19]. The ash content differed significantly among the three fractions (*p* < 0.05), reflecting the distinct distribution of mineral components within the fruit tissues. Powder FC showed the highest ash content, with 5.26%, indicating a high potential for mineral content. The lipid content was low in all fractions (0.79–1.03%) and differed significantly (*p* < 0.05). Although the values varied among fractions, the overall range indicates that lipids represent a minor component of pitomba by-products.

The powders exhibited water activity values below 0.6 (Table 2), indicating that they are unlikely to support microbial growth. Water activity (a_w_) is recognized as one of the main parameters in food preservation, acting as a qualitative indicator of the availability of free water susceptible to physical, chemical, and biological reactions, depending on the processing conditions such as temperature and drying time [20].

The obtained powders exhibited acidic pH values. A significant difference in pH was observed among the fractions (*p* < 0.05). The peel powder (FC) presented the lowest pH values, while the seed powder (FB) showed the highest, with the pulp-with-seed fraction (FA) presenting a value between them. These variations reflect the distinct chemical composition of each fraction. Total carbohydrate content also differed significantly among the fractions (*p* < 0.05), ranging from 84.23% to 86.59%. For the peel fraction (FC), the carbohydrate level (86.59%) was higher than that previously reported for pitomba peel powder (67.82%) [21].

Total dietary fiber differed significantly among the pitomba fractions, ranging from 20.88 to 71.44%, with the peel-derived powder (FC) presenting the highest percentage. In contrast, FA and FB exhibited similar values (approximately 21–22%), indicating that fiber is predominantly concentrated in the peel fraction of the fruit. The higher total dietary fiber content observed in FC may be associated with the structural role of the outer fruit tissue, which generally contains a greater proportion of cell wall–associated materials compared to internal fractions such as pulp and seed. When compared with previous reports, the total dietary fiber content obtained for FC (71.44%) was higher than the crude fiber value reported for pitomba peel powder (52.72%) by Silva et al [21]. The observed difference can be attributed to methodological variation, as crude fiber determination generally yields lower values than enzymatic–gravimetric total dietary fiber methods, such as AOAC 985.29. Overall, these findings reinforce the relevance of the peel fraction as the main fiber reservoir in pitomba residues, highlighting its potential for application in fiber-enriched food formulations.

With regard to caloric value, the powders showed values ranging from 391.48 to 402.23 kcal/100 g. Storck et al. [22] analyzed the nutritional composition of powders derived from fruit residues with different particle sizes, reporting energy values ranging from 205.2 to 389.7 kcal/100 g for grape, 298.6 to 328.3 kcal/100 g for apple, 339.7 to 386.7 kcal/100 g for orange, and 225.6 to 272.7 kcal/100 g for acerola. Although some of these values overlap, the pitomba powders tend to be at the upper end or even exceed the ranges aforementioned.

The titratable acidity (TTA) of pitomba powders ranged from 1.27 to 1.51 g of acid latic per 100 g (wet basis), with the highest value observed for the peel powder (FC). These levels are indicative of the intrinsic acidity of the fruit, which persisted even after drying and milling. In comparison to other tropical fruit powders such as banana peel powder (1.36 g/100 g), lychee peel powder (2.0 g/100 g), pineapple peel powder (2.56 g/100 g) or papaya peel powder (2.62 g/100 g) [23], the pitomba powders exhibited a slightly lower acid content, particularly in the seed fraction (FB). This acidity may influence not only the sensory profile of formulations but also technological aspects, such as buffering capacity, microbial stability, and interaction with leavening agents in baked products [24].

The determination of total soluble solids (TSS), expressed in °Brix, provides an estimate of the concentration of water-soluble compounds in the samples, primarily simple sugars. In the present study, values of 2.0 °Brix were observed for the peel and pulp and seed powders, and 1.5 °Brix for the seed powder. In general, the thermal drying process commonly used in the production of plant-based powders tends to reduce the soluble solids content, due to thermal losses of volatile and unstable compounds and non-enzymatic browning reactions, which consume part of the available reducing sugars [25]. Total soluble solids content varies notably among tropical fruit peel powders: jabuticaba peel powder presents range from 5.1 to 6.3 °Brix [26], papaya peel powders range from 5.8 to 6.0 °Brix, while seed-derived powders typically display lower values, around 1.2 °Brix [27]. These variations underscore the influence of fruit type, maturity stage, and morphological fraction on the retention of soluble sugars, suggesting potential contributions to the natural sweetness of food formulations, particularly in reduced-sugar or sugar correction.

In Table 3, the mineral composition of pitomba powders is detailed, comprising the macroelements potassium (K), calcium (Ca), and magnesium (Mg), together with the microelements iron (Fe), zinc (Zn), and manganese (Mn). Mineral analysis revealed that potassium was the most abundant mineral in the samples, ranging from 692.56 to 1,578.23 mg/100 g, followed by calcium (62.01–354.35 mg/100 g) and magnesium (64.92–91.83 mg/100 g). Among the samples, powder FC showed the most pronounced profile, particularly for calcium (354.35 mg/100 g), corresponding to approximately 35.4% of the recommended daily intake (DRI) for adults and the same proportion for children aged 4–8 years [28]. This contribution is noteworthy given that inadequate calcium intake is associated with an increased risk of osteoporosis and with disturbances affecting bone metabolism as well as cardiovascular, endocrine, and neurological functions [29]. The powders FA and FB contained low calcium (Ca) levels (< 7% of adult DRI), limiting their contribution to Ca intake. Magnesium was more evenly distributed across the samples (64.95–91.83 mg/100 g), being FC accounted for 22.4% of the DRI in man and 29.2% in woman.

For micronutrients, absolute concentrations were lower yet nutritionally meaningful. Iron ranged from 0.47 to 0.84 mg/100 g, with FA contributing 10.5% of the DRI for adult man. Zinc ranged from 1.28 to 1.53 mg/100 g, contributing up to 19.1% of the DRI for woman. Manganese, although present at lower absolute levels (0.31–0.57 mg/100 g), yielded relevant percentage contributions, reaching 38% of the DRI for children in sample FC. These results underscore the importance of considering not only macrominerals but also trace elements, which even in small amounts perform essential metabolic functions in the human. Regarding the absence of copper and the low sodium content (< LOQ*), these findings can be interpreted positively, primarily from a safety standpoint, since excessive intakes of either mineral are associated with adverse health effects. In the case of copper, most individuals meet the dietary intake through other dietary sources. As for sodium, although it is an essential electrolyte for physiological homeostasis, excessive intake is strongly associated with elevated blood pressure [30].

**Table 3:** Total mineral content (mg/100 g) in pitomba powders: pulp with seed (FA), seed (FB), and peel (FC)

Element	Samples			Dietary Reference Intakes (DRIs) Recommended Dietary Allowances and Adequate Intakes, Elements (mg/d) Exception copper (µg/d)		
	FA (mg/100 g)	FB (mg/100 g)	FC (mg/100 g)	Adult man 19–50 years	Adult woman 19–50 years	Children 4–8 years
Calcium	68.57 ± 1.18^b,c^	62.01 ± 2.53^b,c^	354.35 ± 8.87^a^	1000	1000	1000
Magnesium	84.82 ± 1.52^b^	64.95 ± 4.01^c^	91.83 ± 1.62^a^	410	315	130
Iron	0.84 ± 0.06^a,b^	0.47 ± 0.01^c^	0.77 ± 0.01^a,b^	8	18	10
Zinc	1.41 ± 0.03^b^	1.28 ± 0.01^c^	1.53 ± 0.01^a^	11	8	5
Manganese	0.50 ± 0.01^b^	0.31 ± 0.03^c^	0.57 ± 0.03^a^	2.3	1.8	1.5
Copper	< LOQ*	< LOQ*	< LOQ*	900	900	440
Sodium	< LOQ*	7.31 ± 0.54	< LOQ*	1500	1500	1000
Potassium	1466.79 ± 29.46^b^	692.56 ± 16.39^c^	1578.23 ± 30.35^a^	3400	2600	2300

Each value is expressed as mean ± SD for a sample size (*n* = 3). Different letters indicate statistically significant differences among samples according to the applied post hoc test (Tukey’s HSD or Dunn’s test, *p* < 0.05). *< LOQ indicates that the concentration was below the limit of quantification and was not included in the statistical analysis

Dietary Reference Intakes (DRIs) were obtained from the National Academies of Sciences, Engineering, and Medicine (Food and Nutrition Board) [28]

**Fig. 3:**
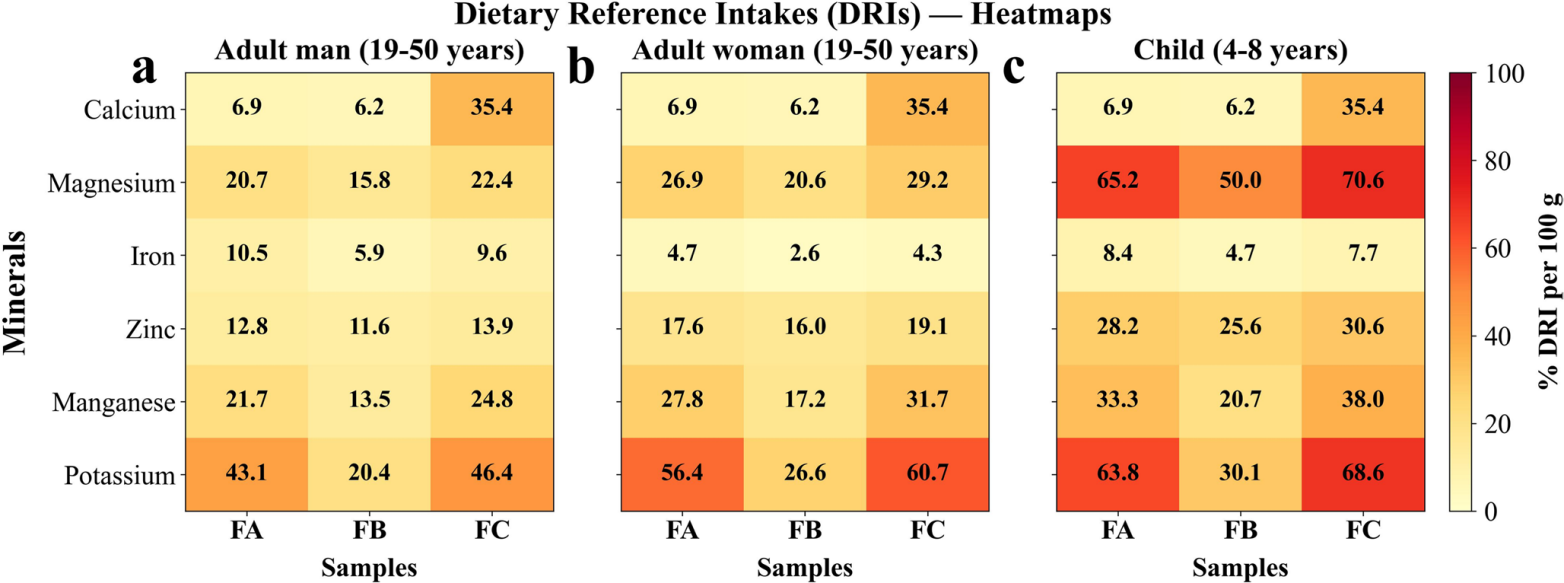
Heatmaps showing the percentage of Dietary Reference Intake (% DRI per 100 g) provided by three samples (FA, FB, and FC) for six essential minerals (calcium, magnesium, iron, zinc, manganese, and potassium) across different population groups: (**a**) Adult man (19–50 years), (**b**) Adult woman (19–50 years), and (**c**) Child (4–8 years)

In aggregate, the heatmaps (Fig. 3) show that the mineral profile of the powders varies according to the population group considered. In adults, the contribution of potassium (≈ 40–46% of the DRI) and calcium in powder FC (35%) stands out. In children, the percentages are even higher, especially in FC, which provided up to 70.6% of the DRI for magnesium, 68.6% for potassium, and 35.4% for calcium. Note that the percentages to the DRI [28] was calculated from the mean contents (mg/100 g of powder), using 100 g portion as basis for comparison with the reference values expressed in mg/day.

Comparing with other fruit residues, Galvão et al. [31] quantified the mineral profile of several commercial fruit powder, and the calcium content in FC (354.35 mg/100 g) exceeded the values reported for green banana (42.3–108.0 mg/100 g), apple (78.5–106.5 mg/100 g), and passion fruit powders (100.6–303.2 mg/100 g), was comparable to grape powder (245.9–369.5 mg/100 g), although lower than orange powder (567.5–581.5 mg/100 g). For magnesium, pitomba values (64.95–91.83 mg/100 g) showed higher values than those of apple (49.6–74.9 mg/100 g), close to grape (61.2–95.8 mg/100 g), for passion fruit (78.6–134.3 mg/100 g), but lower than green banana (110.8–143.7 mg/100 g). For potassium (692.56–1578.23 mg/100 g), pitomba concentrations surpassed apple (687.6–812.6 mg/100 g), was comparable to grape (911.8–1544.1 mg/100 g) and passion fruit (1103.8–2684.9 mg/100 g), but lower than green banana (2100.2–2832.5 mg/100 g).

From a formulation perspective, the high mineral content of the peel powder (FC) supports its use in cereal-based foods, bakery products, and composite powders intended to improve micronutrient levels. Considering its substantial contribution to Dietary Reference Intakes, this fraction may facilitate the formulation of products with enhanced mineral content, depending on serving size and applicable regulations. Additionally, the low sodium content may be advantageous in formulations aligned with sodium-reduction strategies.

### Functional–Technological Properties

**Fig. 4:**
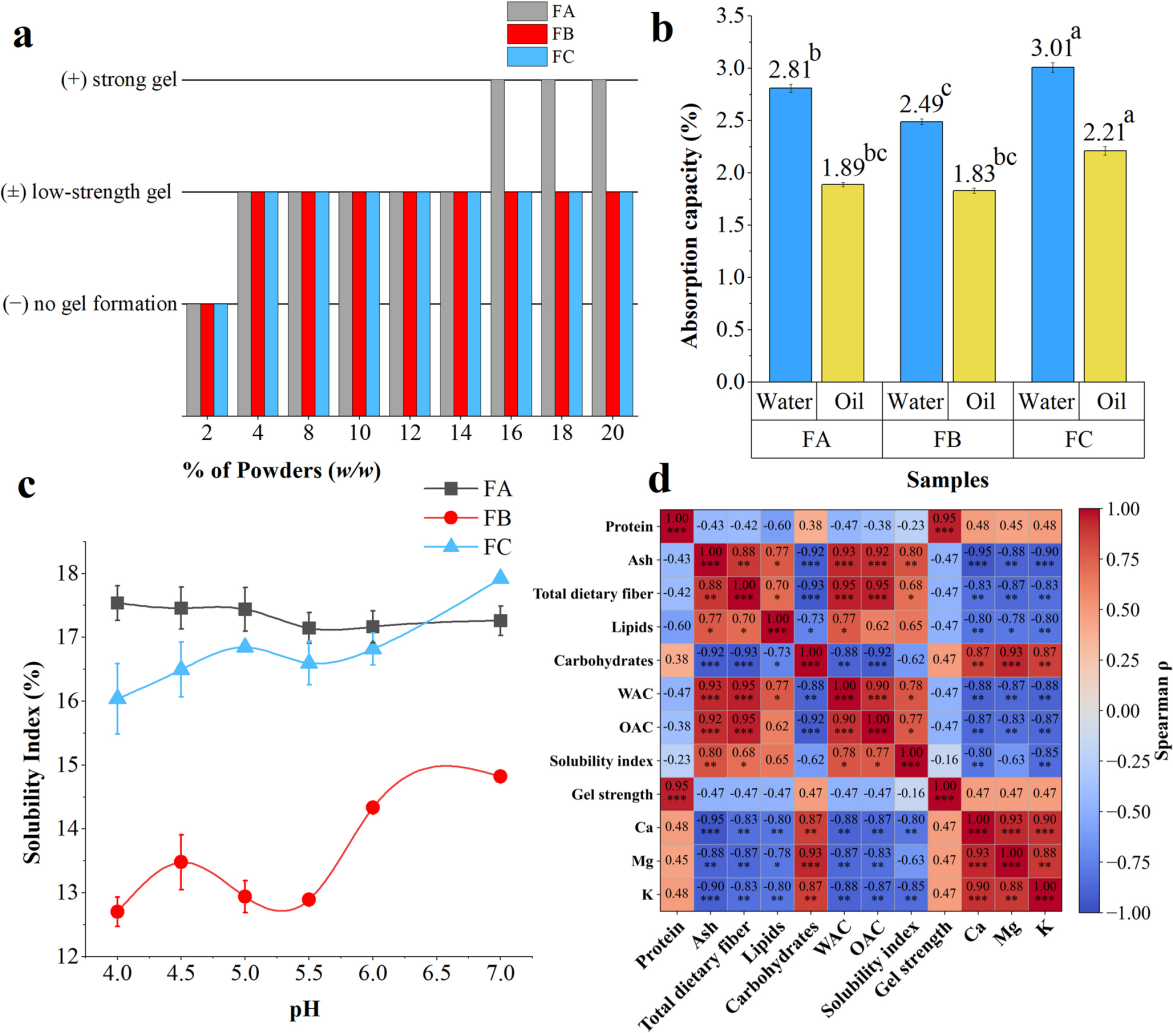
Functional–technological properties of pitomba powders. (**a**) Gelation behavior as a function of powder concentration (% *w/w*), where symbols indicate: no gelation (−); low-strength gel (±); strong gel (+). (**b**) Water absorption capacity (WAC) and oil absorption capacity (OAC) of the powders FA, FB, and FC (blue bars = water; yellow bars = oil), expressed as % ($\:\mathrm{g}\:(\mathrm{w}\mathrm{a}\mathrm{t}\mathrm{e}\mathrm{r}/\mathrm{o}\mathrm{i}\mathrm{l})/\mathrm{g}\:\mathrm{d}\mathrm{r}\mathrm{y}\:\mathrm{m}\mathrm{a}\mathrm{t}\mathrm{t}\mathrm{e}\mathrm{r}$). (**c**) Solubility index (%) of pitomba powders as a function of pH. (**d**) Spearman correlation matrix among chemical composition and functional–technological properties of pitomba powders. Color intensity represents the magnitude and direction of the correlation coefficient (ρ), ranging from − 1 (strong negative correlation) to + 1 (strong positive correlation). Asterisks indicate statistical significance (*p < 0.05; **p < 0.01; ***p < 0.001). Different letters indicate statistically significant differences among samples according to the applied *post hoc* test (Tukey’s HSD or Dunn’s test, *p* < 0.05)

One of the main functional–technological attributes of powders, influencing their application in various foods such as sauces, desserts, soups, and baked goods is the gelation capacity. This technological property is related to the interaction between starches, proteins, and water during heating and cooling processes [32]. The gelation capacity of pitomba powders at different powder concentrations (2% to 20%) is shown in Fig. 4a. In all cases, no gel formation was observed at a concentration of 2%. For the peel powder (FC), gelation began at a concentration of 4%, resulting in low-strength gels. In the powder composed of pulp and seeds (FA), low-strength gels were also detected starting at 4%, while strong gels were formed from 16% onwards. Finally, for the seed powder (FB), gelation was observed starting at 4%, with a strong gel forming at a 20% concentration. Gelation capacity is a relevant technological parameter for structuring semi-solid food systems, particularly in applications such as fillings, sauces, and composite dough formulations.

As observed in Fig. 4b, the water absorption capacity of pitomba powders was higher than their oil absorption capacity, regardless of the fraction evaluated. FC exhibited the highest water absorption capacity (3.01 g/g) and oil absorption (2.21 g/g) among the evaluated powders, differing statistically from the others (*p* < 0.05) and suggesting that its composition or processing conferred greater capacity for retaining both polar and non-polar liquid.

From a technological standpoint, powders with high water absorption values are desirable for products such as breads, cakes, and pasta, in which moisture retention improves the texture and yield of the final product. In turn, powders with higher oil absorption are important for meat products, sauces, and fillings, contributing to juiciness and flavor enhancement [33].

In summary, the functional–technological behavior observed for pitomba powders is consistent with trends reported for powders obtained from fruit by-products, particularly those derived from peels and seeds. In such systems, gelation capacity is strongly concentration-dependent, with gel formation occurring only at moderate to high solid contents, while water absorption capacity generally exceeds oil absorption capacity due to the predominance of hydrophilic components, such as dietary fiber. Comparable fraction-dependent behavior has also been described for seed powders from fruits belonging to the Sapindaceae family, supporting the technological relevance of pitomba powders within this botanical group [34].

The solubility index evaluated in this study reflects the total water-soluble fraction of the powder matrix, not specifically protein solubility. Therefore, the pH-dependent behavior observed (Fig. 4c) reflects the combined contribution of soluble carbohydrates, low-molecular-weight compounds, organic acids, and mineral constituents. The peel powder (FC) showed a gradual increase in solubility toward neutral pH, suggesting greater release or dispersion of matrix components under less acidic conditions. The seed powder (FB) exhibited more pronounced variation across the pH range, possibly indicating structural differences in its matrix composition. In contrast, the pulp with seed powder (FA) displayed relatively stable solubility values, suggesting a more homogeneous distribution of soluble constituents.

Because the solubility index measured here refers to total matrix solubility, the absence of a sharp minimum solubility region is expected and does not necessarily reflect protein isoelectric behavior. Instead, the results indicate that solubility is governed by the complex interaction of carbohydrates, fiber, and mineral components typical of fruit-derived powders. From a technological standpoint, solubility is a key functional parameter for ingredients intended for beverage systems and reconstituted products, as it directly affects dispersion efficiency, sedimentation behavior, and overall product stability [35]. The pH-dependent variations observed in the present study indicate that solubility is influenced by environmental conditions, which may impact ingredient performance in different food matrices.

Although the differences across the evaluated pH range were moderate, such variations could affect dispersion characteristics in liquid and semi-liquid systems. These findings suggest that the functional performance of pitomba powders may depend on formulation pH, warranting further investigation in model food systems.

Spearman correlation analysis (Fig. 4d) demonstrated distinct compositional–functional associations. Total dietary fiber showed strong positive correlations with WAC (*ρ* = 0.95, *p* < 0.001) and OAC (*ρ* = 0.95, *p* < 0.001), while ash content was similarly associated with WAC (*ρ* = 0.93, *p* < 0.001) and OAC (*ρ* = 0.92, *p* < 0.001), indicating that hydration and oil retention behavior are associated with the structural composition of the fractions. The solubility index was moderately to strongly correlated with ash (*ρ* = 0.80, *p* < 0.01), total dietary fiber (*ρ* = 0.68, *p* < 0.05), WAC (*ρ* = 0.78, *p* < 0.05), and OAC (*ρ* = 0.77, *p* < 0.05), indicating that dispersibility tends to co-vary with hydration-related properties and overall compositional characteristics of the fractions. Conversely, protein content was strongly correlated with gel strength (*ρ* = 0.95, *p* < 0.001), suggesting that gel formation is primarily influenced by protein concentration rather than by fiber-related hydration capacity. Carbohydrates, calculated by difference, exhibited strong inverse correlations with total dietary fiber (*ρ* = −0.93, *p* < 0.001) and hydration-related properties, reflecting the compositional distribution among fractions rather than independent functional effects. Other correlations were considered secondary or structurally related to the compositional distribution among fractions.

## Conclusions

This study provided a systematic nutritional and functional–technological characterization of powders obtained from different morphological fractions of *Talisia esculenta*, revealing significant compositional and functional differences among pulp with seed, seed, and peel fractions. The peel powder showed the highest mineral contents, with notable contributions to Dietary Reference Intakes (DRIs), particularly for potassium, calcium, and magnesium, and dietary fiber percentage, while all fractions presented good technological properties, including water and oil absorption capacities and gelation behavior, supporting their potential use as functional food ingredients. These findings demonstrate that pitomba residues can be considered nutrient-dense raw materials with promising applicability in food enrichment and in the development of value-added products, contributing to the sustainable utilization of a Brazilian native fruit. Future research should focus on optimizing processing conditions to improve functional performance, as well as evaluating the incorporation of these powders into different food matrices. Further analytical approaches, including more detailed characterization of bioactive compounds and other minor constituents, may provide additional insight into their nutritional relevance and technological potential.

## Supplementary Information

Below is the link to the electronic supplementary material.

Supplementary Material 1

## Data Availability

The datasets created during this study can be obtained from the corresponding author upon reasonable request.

## References

[1] Sviech F, Ubbink J, Ana Silvia P (2022) Potential for the processing of Brazilian fruits - A review of approaches based on the state diagram. LWT 156:113013. 10.1016/j.lwt.2021.113013

[2] Fraga L, Nascimento AKS, Oliveira BP Aragão Ana Mara de Oliveira e Silva, Elma Regina Silva de Andrade Wartha, Leandro Bacci, Luciana Pereira Lobato, and Izabela Maria Montezano de Carvalho (2020). Physico-chemical characterization of the pulp and peel of Brazilian Pitomba (Talisia esculenta (A. St.-Hill.) Radlk). Res Soc Dev 9: e122911774–e122911774. 10.33448/rsd-v9i1.1774

[3] Guarim Neto, Germano SR, Santana, Valdete Bezerra J, Silva (2003) Repertório botânico da Pitombeira (Talisia esculenta (A. ST.-HIL.) Radlk. - Sapindaceae). Acta Amazonica 33:237–242. Instituto Nacional de Pesquisas da Amazônia10.1590/1809-4392200332242

[4] Sagar N, Alok S, Pareek S, Sharma EM, Yahia, and Maria Gloria Lobo (2018) Fruit and Vegetable Waste: Bioactive Compounds, Their Extraction, and Possible Utilization. Compr Rev Food Sci Food Saf 17:512–531. 10.1111/1541-4337.12330 33350136 10.1111/1541-4337.12330

[5] Jara Ramirez Victoria N, Gonzales Zaira O, Aguinaga Danny ILA Munoz Ccuro Felipa, Roman Perez Hitler, and Benites-Alfaro Elmer (2023). Circular Economy: Use of Fruit Waste to Obtain Bioplastics. Chem Eng Trans 100: 103–108. 10.3303/CET23100018

[6] Zarzycki P, Wirkijowska A (2024) and Urszula Pankiewicz. Functional Bakery Products: Technological, Chemical and Nutritional Modification. Applied Sciences 14. Multidisciplinary Digit Publishing Inst 12023. 10.3390/app142412023

[7] Nainggolan E, Alga J, Banout, and Klara Urbanova (2024) Recent Trends in the Pre-Drying, Drying, and Post-Drying Processes for Cassava Tuber: A Review. Foods 13:1778. 10.3390/foods13111778. Multidisciplinary Digital Publishing Institute 38891006 10.3390/foods13111778 PMC11171685

[8] Campos DéboraA, Gómez-García R, Vilas-Boas AA, Madureira AR, Maria Manuela Pintado (2020) Management of Fruit Industrial By-Products—A Case Study on Circular Economy Approach. Molecules 25 Multidisciplinary Digit Publishing Inst 320. 10.3390/molecules25020320 10.3390/molecules25020320 PMC7024247 31941124

[9] Castro DS, De IDS, Moreira FCD, Sousa (2021) Physical, chemical and rheological properties of pitomba (Talisia esculenta) seed starch and its application as a thickener and stabilizer in ketchup. Aust J Crop Sci 842–849. Wilton Pereira Da Silva, Josivanda Palmeira Gomes, Alexandre José De Melo Queiroz, Cleide Maria Diniz Pereira Da Silva E Silva, and Bruno Adelino De Melo 10.21475/ajcs.21.15.06.p2981

[10] Souza, Pâmelada, Leila Larisa Medeiros Marques (2024) Silva Flávia Aparecida Reitz Cardoso, Mirela Vanin dos Santos Lima, Stênio Cristaldo Heck, and. Extraction of bioactive compounds from peel and seeds of pitomba (Talisia esculenta) using eutectic solvents. Int J Food Sci Technol 59: 9060–9071. 10.1111/ijfs.17460

[11] Aschemann-Witzel J, Bizzo HR, Ana Carolina S, Doria Chaves AF, Amauri Rosenthal (2023) Faria-Machado Antonio Gomes Soares, Marcos José de Oliveira Fonseca, Ulla Kidmose, and. Sustainable use of tropical fruits? Challenges and opportunities of applying the waste-to-value concept to international value chains. Critical Reviews in Food Science and Nutrition 63. Taylor & Francis: 1339–1351. 10.1080/10408398.2021.1963665 10.1080/10408398.2021.1963665 34382890

[12] Vega-Gálvez A, Scala KD, Rodríguez K, Lemus-Mondaca R, Miranda M, López J, and Mario Perez-Won (2009) Effect of air-drying temperature on physico-chemical properties, antioxidant capacity, colour and total phenolic content of red pepper (*Capsicum annuum*, L. var. Hungarian). Food Chem 117:647–653. 10.1016/j.foodchem.2009.04.066

[13] Medeni Maskan (2006) Effect of thermal processing on tristimulus colour changes of fruits. Stewart Postharvest Rev 2:1–8. 10.2212/spr.2006.5.10

[14] Abedin M, Jaynal MR, Sarker SA, Siyam, Farzana T (2025) Exploring the effect of pumpkin, soy, and sweet potato flours on soup’s nutrition, antioxidant capacity, and sensory attributes. Food Chem Adv 7 Elsevier BV 101019. 10.1016/j.focha.2025.101019

[15] ANVISA - Brazilian Health Regulatory Agency (2005) *Resolution RDC No. 263, September 22, 2005*. Resolution. Regulamento técnico para produtos de cereais, amidos, farinha e farelos. Brazil, Brasília, DF, Brazil

[16] Soares LV, Ferreira, Cristiane Daliassi Ramos de Souza (2024) Análise do teor de umidade como parâmetro de qualidade na produção de farinhas de trigo. Res Soc Dev 13:e135131047191–e135131047191. 10.33448/rsd-v13i10.47191

[17] Araújo, Ana Maria de Souza (2024) Aproveitamento dos resíduos do fruto da pitombeira (Talisia esculenta) para elaboração de subproduto. Universidade Federal de Campina Grande

[18] Santos N, Carlos RLJ Almeida Tamires dos Santos Pereira, Anna Paula Rocha de Queiroga, Virgínia Mirtes de Alcântara Silva, Deborah Silva do Amaral, Renata Duarte Almeida, Victor Herbert de Alcântara Ribeiro, Eliélson Rafael Barros, and Lucas Rodolfo Inácio da Silva. 2020. Modelagem matemática aplicada a cinética de secagem das cascas de pitomba (Talisia esculenta). Res Soc Dev 9: e46921986–e46921986. 10.33448/rsd-v9i2.1986

[19] Ministério da Agricultura, Pecuária e Abastecimento (2005) Instrução Normativa n^o^ 8, de 2 de junho de 2005 – Regulamento Técnico para Farinha de Trigo. Governo Federal do Brasil

[20] dos Carneiro S, Andreola WK Carlos Eduardo de Farias Silva, Brígida Maria Villar da Gama, Rosana Correia Vieira Albuquerque, Jeniffer Mclaine Duarte de Freitas, and Johnnatan Duarte de Freitas. 2024. Agglomeration Process of Spirulina platensis Powder in Fluidized Bed Improves Its Flowability and Wetting Capacity. ACS Food Sci Technol 4 Am Chem Soc : 3120–3134. 10.1021/acsfoodscitech.4c00745

[21] Silva GM, Da, DAS CASCAS DE CUPUAÇU E PITOMBA (2021) Remerson Joaquim De Araújo Moreira, José Elias Cândido, Izaqueu Rodrigues Da Silva, Bruno Anderson De Morais, Marcos Juliano Gouveia, Tonny Cley Campos Leite, and Amanda Reges De Sena. OBTENÇÃO DE FARINHAS : ANÁLISE BROMATOLÓGICA E FITOQUÍMICA. In *Extensão Rural: Práticas e Pesquisas Para o Fortalecimento da Agricultura Familiar - Volume 1*, Robson José De Oliveira, 1st ed., 431–449. Editora Científica Digital. 10.37885/210102768

[22] Storck Cátia, Regina C, Basso FR, Favarin, Alessandra Cristina R (2015) Qualidade microbiológica e composição de farinhas de resíduos da produção de suco de frutas em diferentes granulometrias. *Brazilian Journal of Food Technology* 18. Instituto de Tecnologia de Alimentos - ITAL: 277–284. 10.1590/1981-6723.1615

[23] Silva Jéssyca, Santos DW, Ortiz ER, Asquieri, Damiani C (2020) Physicochemical and technological evaluation of flours made from fruit co-products for use in food products. Res Soc Dev 9:e192932742–e192932742. 10.33448/rsd-v9i3.2742

[24] Mariotti M, Garofalo C, Aquilanti L, Osimani A, Fongaro L, Tavoletti S, Hager A-S, Clementi F (2014) Barley flour exploitation in sourdough bread-making: A technological, nutritional and sensory evaluation. LWT - Food Sci Technol 59:973–980. 10.1016/j.lwt.2014.06.052

[25] Schutte M, Hayward S, and Marena Manley (2024) Nonenzymatic Browning and Antioxidant Properties of Thermally Treated Cereal Grains and End Products. J Food Biochem 2024:3865849. 10.1155/2024/3865849

[26] Resende LaísM, Leandro S, Oliveira, Franca AS (2020) Characterization of jabuticaba (Plinia cauliflora) peel flours and prediction of compounds by FTIR analysis. LWT 133:110135. 10.1016/j.lwt.2020.110135

[27] Santos Cláudia Mendes dos Celeste Maria Patto de Abreu, Juliana Mesquita Freire, Estela de Rezende Queiroz, and Marcelle Mendes Mendonça (2014). Chemical characterization of the flour of peel and seed from two papaya cultivars. Food Sci Technol 34. Sociedade Brasileira de Ciência e Tecnologia de Alimentos: 353–357. 10.1590/fst.2014.0048

[28] National Academies of Sciences, Oria EM, Harrison M, Stallings VA (2019) Dietary Reference Intakes (DRIs): Recommended Dietary Allowances and Adequate Intakes, Elements, Food and Nutrition Board, National Academies. Text. National Academies Press (US). March 5 30844154

[29] Shlisky J, Mandlik R, Askari S, Abrams S, Belizan JM, Bourassa MW, Cormick G et al (2022) Calcium deficiency worldwide: prevalence of inadequate intakes and associated health outcomes. Ann N Y Acad Sci 1512:10–28. 10.1111/nyas.14758 35247225 10.1111/nyas.14758 PMC9311836

[30] Hunter RW, Dhaun N, Bailey MA (2022) The impact of excessive salt intake on human health. Nature Reviews Nephrology 18 Nat Publishing Group 321–335. 10.1038/s41581-021-00533-0 10.1038/s41581-021-00533-0 35058650

[31] Galvão F, Pinto E, Martins ZE, Almeida AA, Isabel MPLVO, Ferreira (2023) Nutritional composition and minerals bioaccessibility of commercial fruit flours. J Food Meas Charact 17:2547–2554. 10.1007/s11694-022-01747-x. Vanderlei Aparecido de Lima, and Maria Lurdes Felsner

[32] Ma Y, Guo L, Chen Y, Wang SYL, Zhao K, Li H et al (2025) Quinoa flour addition improves the rheological properties and resistant starch content of wheat dough: From the view of starch physicochemical properties and starch-protein interaction. LWT 223:117804. 10.1016/j.lwt.2025.117804

[33] De Angelis D, Latrofa V, Squeo G, Pasqualone A, Summo C (2024) Techno-functional, rheological, and chemical properties of plant-based protein ingredients obtained with dry fractionation and wet extraction. Curr Res Food Sci 9:100906. 10.1016/j.crfs.2024.100906 39555018 10.1016/j.crfs.2024.100906 PMC11565420

[34] Eiamwat J, Wanlapa S (2016) and Sukit Kampruengdet. Physicochemical Properties of Defatted Rambutan (Nephelium lappaceum) Seed Flour after Alkaline Treatment. *Molecules* 21. Multidisciplinary Digital Publishing Institute: 364. 10.3390/molecules21040364 10.3390/molecules21040364 PMC6273815 27043520

[35] Ren Y, Jia F, Li D (2024) Ingredients, structure and reconstitution properties of instant powder foods and the potential for healthy product development: a comprehensive review. Food Funct 15:37–61. The Royal Society of Chemistry10.1039/D3FO04216B 38059502 10.1039/d3fo04216b

