# Supplementary material for: Valorization of a Low-Pulp Brazilian Native Fruit Pitomba (Talisia esculenta Radlk) Through the Production of Nutritive Powders with the Whole Fruit
Source: Plant Foods Hum Nutr. 2026 Apr 21;81(2):48. doi: 10.1007/s11130-026-01493-1 (PMC13100009; doi:10.1007/s11130-026-01493-1)
Supplement: Supplementary file 1 — Supplementary Material 1 [file 11130_2026_1493_MOESM1_ESM.docx]

**Supplementary Material**

**Valorization of a low-pulp Brazilian Native Fruit Pitomba (*Talisia esculenta* Radlk) through the Production of Nutritive Powders with the Whole Fruit**

Wanderson dos Santos Carneiro^a^, Carlos Eduardo de Farias Silva^a*^, Kaciane Andreola^b^, Ana Silvia Prata^c^

^a^ Technology Center, Federal University of Alagoas, Maceió, Alagoas, 57072-970, Brazil. ^b^ Institute of Food Technology, Campinas, São Paulo, 13070-178, Brazil. ^c^ Department of Food Engineering and Technology, School of Food Engineering (FEA), State University of Campinas (UNICAMP), Campinas, São Paulo, Brazil

*corresponding-author: carlos.eduardo@ctec.ufal.br

## **Materials and Methods**

**Sample acquisition and preparation**

The samples were collected in the municipality of Água Branca, located in the state of Paraíba, in the Northeast region of Brazil (Latitude: 7° 30′ 54″ S, Longitude: 37° 38′ 28″ W) (Brazilian SisGen access number A03A93C). After collection, the samples were sanitized by immersion in a chlorinated water solution at 100 ppm for 15 minutes, then rinsed under running water to remove residual chlorine. Subsequently, the fruits were manually peeled and pulped using gloves, and then subdivided into three categories: pulp with seed mixture (FA), seed (FB), and peel (FC) (Fig. 1). The pulp adhered to the seed was removed manually using a handheld vegetable peeler. The tool was applied around the seed with gentle rotational movements, allowing mechanical abrasion to detach and eliminate the remaining pulp before further processing. Each fraction was then packaged in airtight polyethylene bags and stored at −20 °C until further analyses.

**Preparation of Pitomba Powders**

The raw materials were dried in a forced-air circulation (EST.420 4D, Ethik Technology, Brazil), using stainless steel trays, with air circulating oven at 50 °C. Drying times varied according to morphological fraction: 48 hours for the seed and peel, and 60 hours for the seed–pulp mixture. After drying, the samples were ground using a knife mill and then further milled in an analytical grinder (Model A11, IKA, Germany). The resulting material was sieved using a 45 mesh (355 µm) sieve supplied by Brozinox (São Paulo, Brazil).

**Color Analysis**

Color was determined by the CIE method using a spectrophotometer (CM-600d, Konica Minolta, Japan). Parameters *L** (lightness), *a** (red–green), and *b** (yellow–blue) were recorded in triplicate using illuminant D65 and a 10° standard observer. The total color difference (ΔE*) was calculated according to the CIE76 equation (Equation 1) [1]:

| ${\Delta E}^{*}=\sqrt{{({\Delta L}^{*})}^{2}+{({\Delta a}^{*})}^{2}+{({\Delta b}^{*})}^{2}}$ | (Equation 1) |
| --- | --- |

where *ΔL**, *Δa**, and *Δb** represent the differences between the *L**, *a**, and *b** coordinates of two samples. Pairwise comparisons were performed for FA–FB, FA–FC, and FB–FC using the mean *L**, *a**, and *b** values of each sample.

**Physical and chemical Characterization**

The proximate composition was determined according to AOAC [2]. Crude protein was determined using the Kjeldahl method (AOAC 984.13), with a nitrogen conversion factor of 6.25. The total lipid content was determined using the method described by Bligh and Dyer [3] and quantified by gravimetry. The quantitative determination of total ashes was carried out by the residue combustion method at 500 °C according to AOAC 984.13. Moisture content was determined by direct drying in an oven at 105 °C following AOAC 925.10.

The carbohydrate content was determined by the difference of the aforementioned components (proteins, lipids, and ash) considering dry basis, following AOAC 986.25 (Equation 2). The energy value (kcal/100g) was determined using the Atwater Equation (Equation 3), which considers the average values obtained for each macronutrient from conventional methods [4].

| Total Carbohydrates (%) = 100 – (%Protein + %Lipids + %Ash) | (Equation 2) |
| --- | --- |
| Caloric value (kcal/100g) = Protein x 3.87 + Fat x 8.37 + Carbohydrate x 4.11 | (Equation 3) |

Total dietary fiber was as determined according to method AOAC 985.29. Water activity was measured by direct reading using a hygrometer (AQUAlab, Decagon 4TE, USA) at 25 °C. The pH, total titratable acidity (TTA) expressed as g lactic acid per 100 g of sample, and total soluble solids (ºBrix) were analyzed according to the protocols of the AOAC 981.12, 942.15, and 932.12, respectively.

Mineral content was determined according to the procedure described by Costa-Santos et al. [5], with adaptations. Approximately 0.60 g of each sample was digested in an open-block system using concentrated nitric acid (4 mL; Merck, Darmstadt, Germany) at 110 °C for 2 h, followed by the addition of concentrated nitric acid (4 mL) and hydrogen peroxide (2 mL; Merck, Darmstadt, Germany). Digestion was then continued at 130 °C for an additional 2 h. The digests were filtered, diluted with ultrapure water, and analyzed using an atomic absorption spectrometer (AAnalyst 200, PerkinElmer, Norwalk, CT, USA) equipped with deuterium background correction. Quantification was performed in an air–acetylene flame at ~2000 °C, with Fe, Ca, Cu, Mg, Mn, and Zn measured using hollow cathode lamps at their specific wavelengths, and K determined by atomic emission. All analyses were conducted in triplicate.

**Functional–technological properties**

Gelling properties were assessed according to Sathe and Salunkhe [6]. Sample suspensions (2–20% w/w) were prepared in 5 mL distilled water, heated at 90 °C for 1 h, cooled, and refrigerated at 4 °C for 2 h. The minimum concentration required for gelation was determined as the one where the formed gel remained stable in its initial position without flowing down the wall. Gel strength was qualitatively classified as follows: no gel (-) (complete flow after inversion), low-strength gel (±) (partial flow or deformation along the tube wall), and strong gel (+) (no observable flow, maintaining structural integrity after inversion).

The determination of water absorption capacity (WAC) and oil absorption capacity (OAC) where determined following Abu [7], with modifications. One gram of the powder was mixed with 10 mL of distilled water (for WAC) or soybean oil (for OAC), vortexed for 2 minutes and centrifuged at 3000×g for 20 minutes. Solubility was determined at different pH values following the method described in Silva et al. [8] with some modifications. A 2.5% (*w/v*) aqueous dispersion was prepared and adjusted at four different pH (4, 4.5, 5, 5.5, 6, 7) by using 0.1 N NaOH or HCl.

**Statistical analysis**

Statistical analysis was performed using one-way analysis of variance (ANOVA), after verification of normality of residuals (Shapiro–Wilk test) and homogeneity of variances (Brown–Forsythe test). When the assumptions were met, Tukey’s HSD test was applied for multiple comparisons of means. In cases where assumptions were not satisfied, the Kruskal–Wallis test followed by Dunn’s post hoc test was used. A significance level of 5% (*α* = 0.05) was adopted. Results are presented as mean ± standard deviation (*n* = 3), and different letters indicate statistically significant differences among groups. Additionally, Spearman’s rank correlation analysis was performed to evaluate associations between compositional parameters (proximate composition and mineral content) and physicochemical/functional properties. Correlation coefficients (ρ) and corresponding p-values were calculated, and the results were presented as a correlation matrix.

**Computational analyses in Python**

Statistical and graphical analyses were performed using Python (v. 3.13.5). One-way analysis of variance (ANOVA) was conducted with the *statsmodels* library, and multiple comparisons were performed using Tukey’s honestly significant difference (HSD) test implemented in *statsmodels* and *scikit-posthocs*. The analyses were executed within the Spyder IDE (v. 6.0.7).

**References**

1. Vega-Gálvez, Antonio, Karina Di Scala, Katia Rodríguez, Roberto Lemus-Mondaca, Margarita Miranda, Jessica López, and Mario Perez-Won. 2009. Effect of air-drying temperature on physico-chemical properties, antioxidant capacity, colour and total phenolic content of red pepper (*Capsicum annuum*, L. var. Hungarian). *Food Chemistry* 117: 647–653. https://doi.org/10.1016/j.foodchem.2009.04.066.

2. AOAC. 2005. Official Methods of Analysis of AOAC International. In *Official Methods of Analysis*, 18th ed. Gaithersburg, MD, USA: AOAC International.

3. Bligh, E. G., and W. J. Dyer. 1959. A rapid method of total lipid extraction and purification. *Canadian Journal of Biochemistry and Physiology* 37: 911–917. https://doi.org/10.1139/o59-099.

4. Merrill, A. L., and B. K. Watt. 1973. *Energy Values of Foods: Basis and Derivation*. Agriculture Handbook 74. Washington, DC: United States Department of Agriculture.

5. Costa-Santos, Augusto César, Ana Paula Rebellato, Eduardo Adilson Orlando, and Juliana Azevedo Lima Pallone. 2024. Characterization and estimation of the bioaccessibility of essential elements in organic milk by INFOGEST protocol. *Food Chemistry* 433: 137327. https://doi.org/10.1016/j.foodchem.2023.137327.

6. Sathe, S. K., and D. K. Salunkhe. 1981. Functional Properties of the Great Northern Bean (Phaseolus vulgaris L.) Proteins: Emulsion, Foaming, Viscosity, and Gelation Properties. *Journal of Food Science* 46: 71–81. https://doi.org/10.1111/j.1365-2621.1981.tb14533.x.

7. Abu, Michael Sunday. 2023. Comparative evaluation of cassava composite flours and bread. *Asian Journal of Natural Product Biochemistry* 21. https://doi.org/10.13057/biofar/f210103.

8. Silva, Jéssyca Santos, Daniela Weyrich Ortiz, Eduardo Ramirez Asquieri, and Clarissa Damiani. 2020. Physicochemical and technological evaluation of flours made from fruit co-products for use in food products. *Research, Society and Development* 9: e192932742–e192932742. https://doi.org/10.33448/rsd-v9i3.2742.
